# Supplementary material for: Cu@Pd/C with Controllable Pd Dispersion as a Highly Efficient Catalyst for Hydrogen Evolution from Ammonia Borane
Source: Nanomaterials (Basel). 2020 Sep 16;10(9):1850. doi: 10.3390/nano10091850 (PMC7558311; doi:10.3390/nano10091850)
Supplement: Supplementary file 1 [file nanomaterials-10-01850-s001.pdf]

Supporting information

# Cu@Pd /C with Controllable Pd Dispersion as a Highly Efficient Catalyst for Hydrogen Evolution from Ammonia Borane

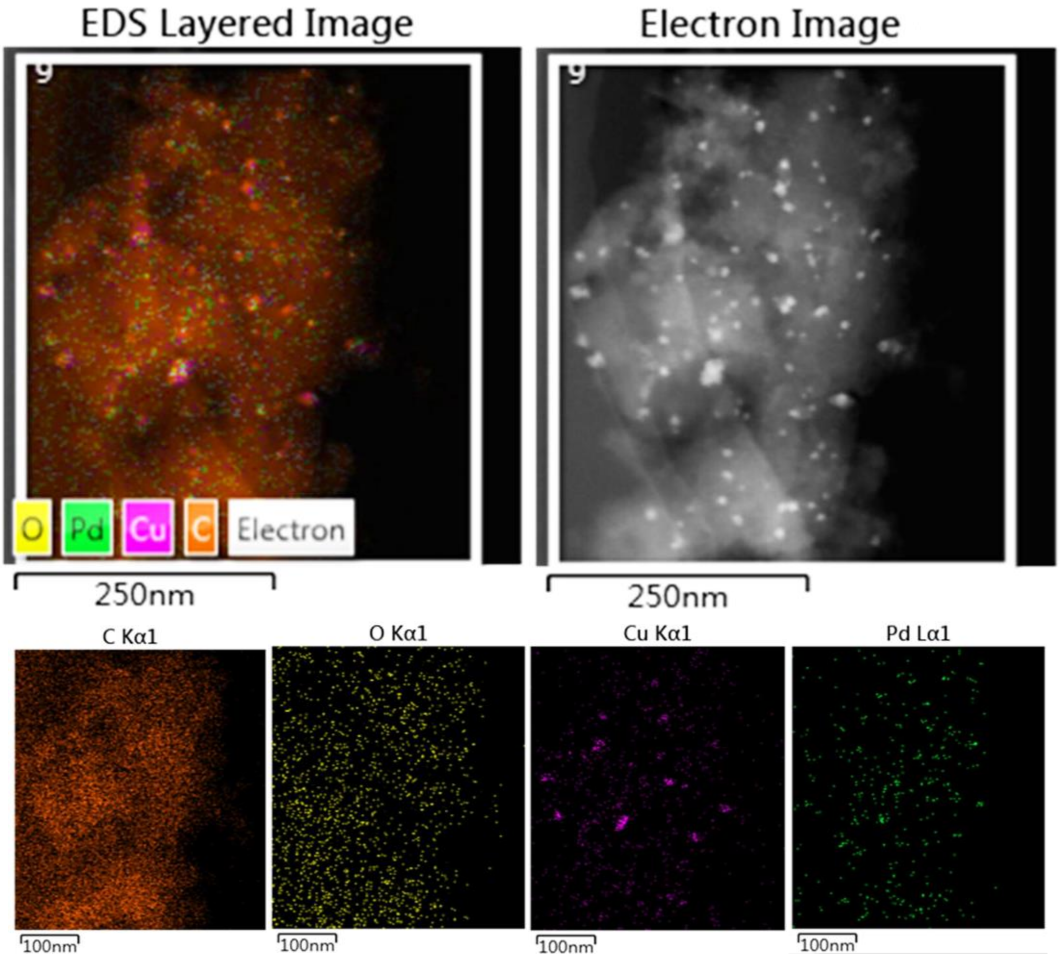

Figure S1. EDS mapping images of $Cu@Pd_{0.75}/C$-320.

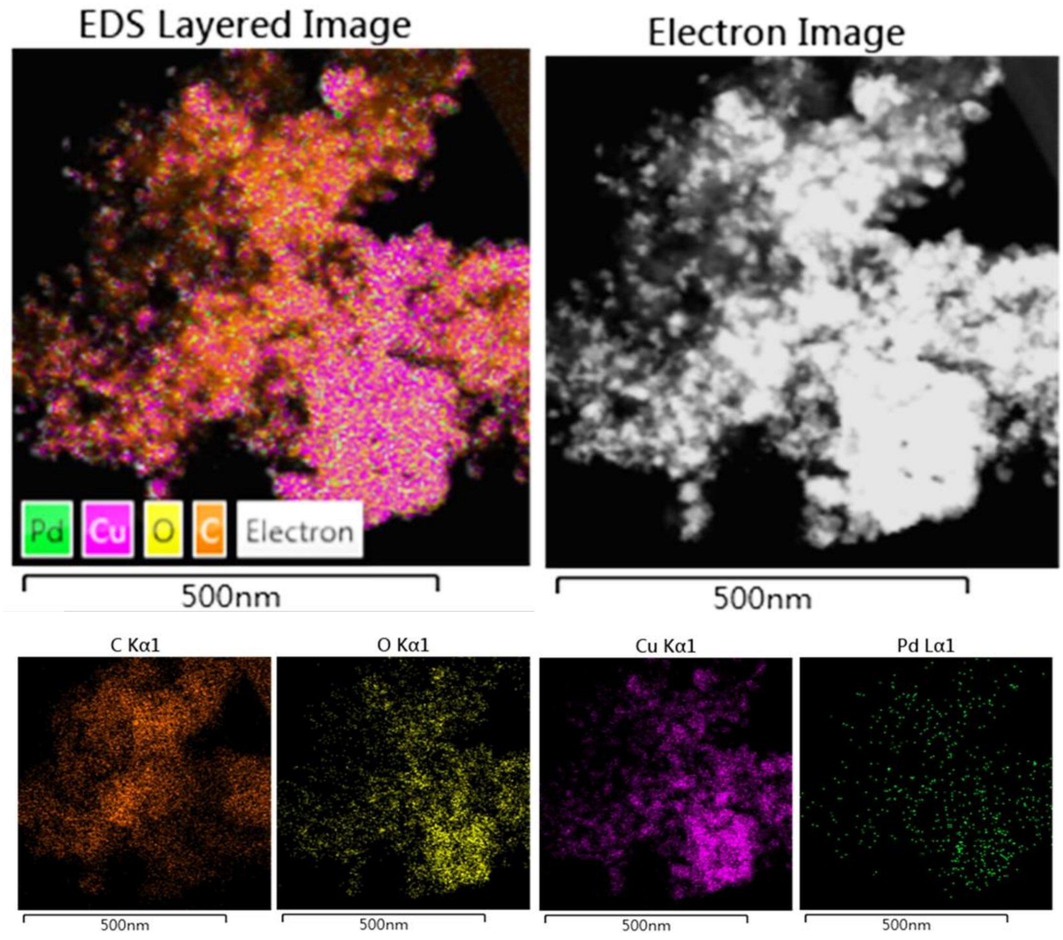


Figure S2. EDS mapping images of Cu@$Pd_{1.0}$/C-320.

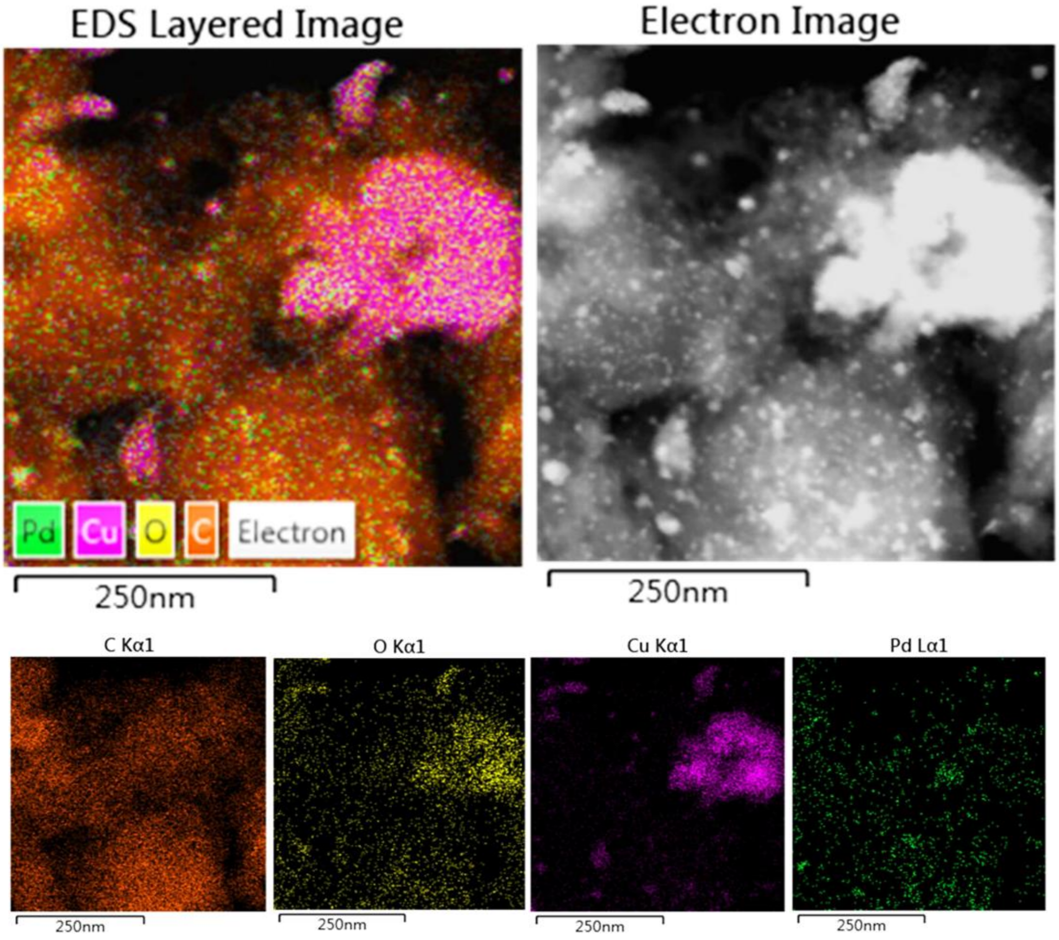


Figure S3. EDS mapping images of Cu@$Pd_{2.0}$/C-320.

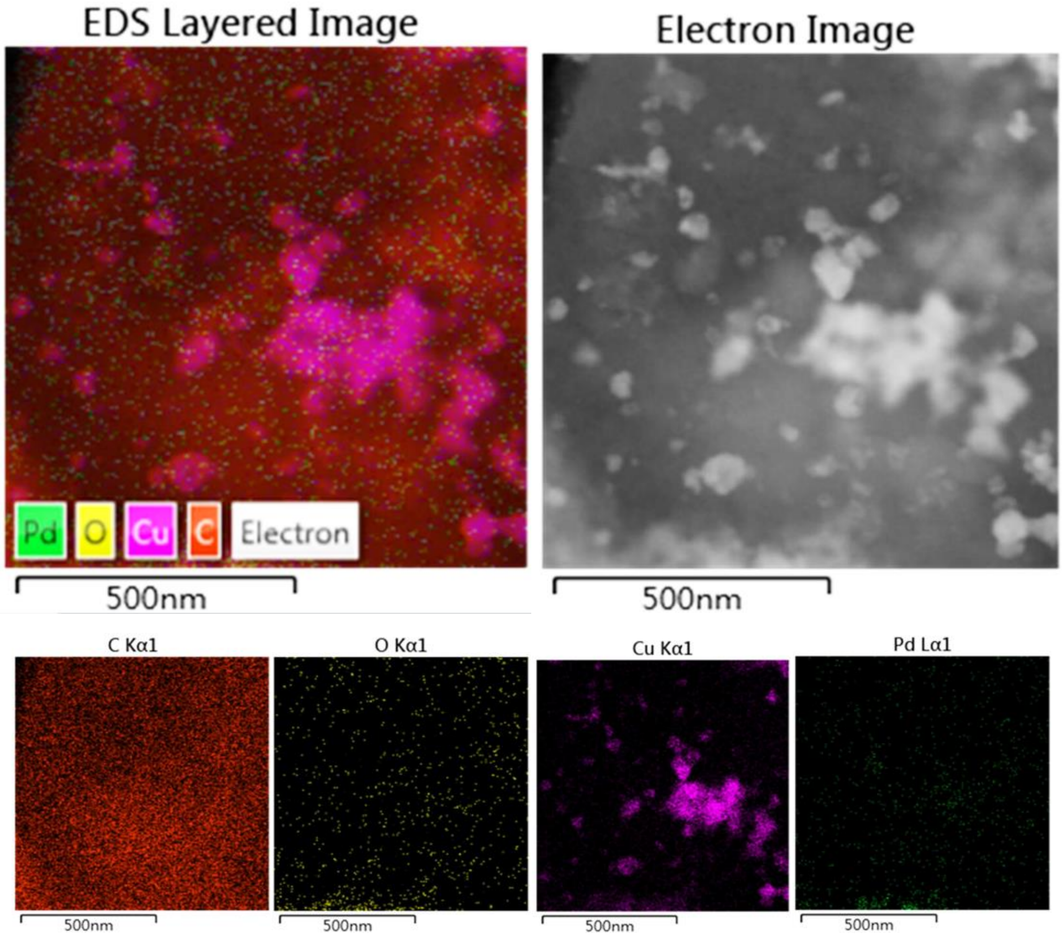


Figure S4. EDS mapping images of Cu@$Pd_{0.5}$/C-320-R.

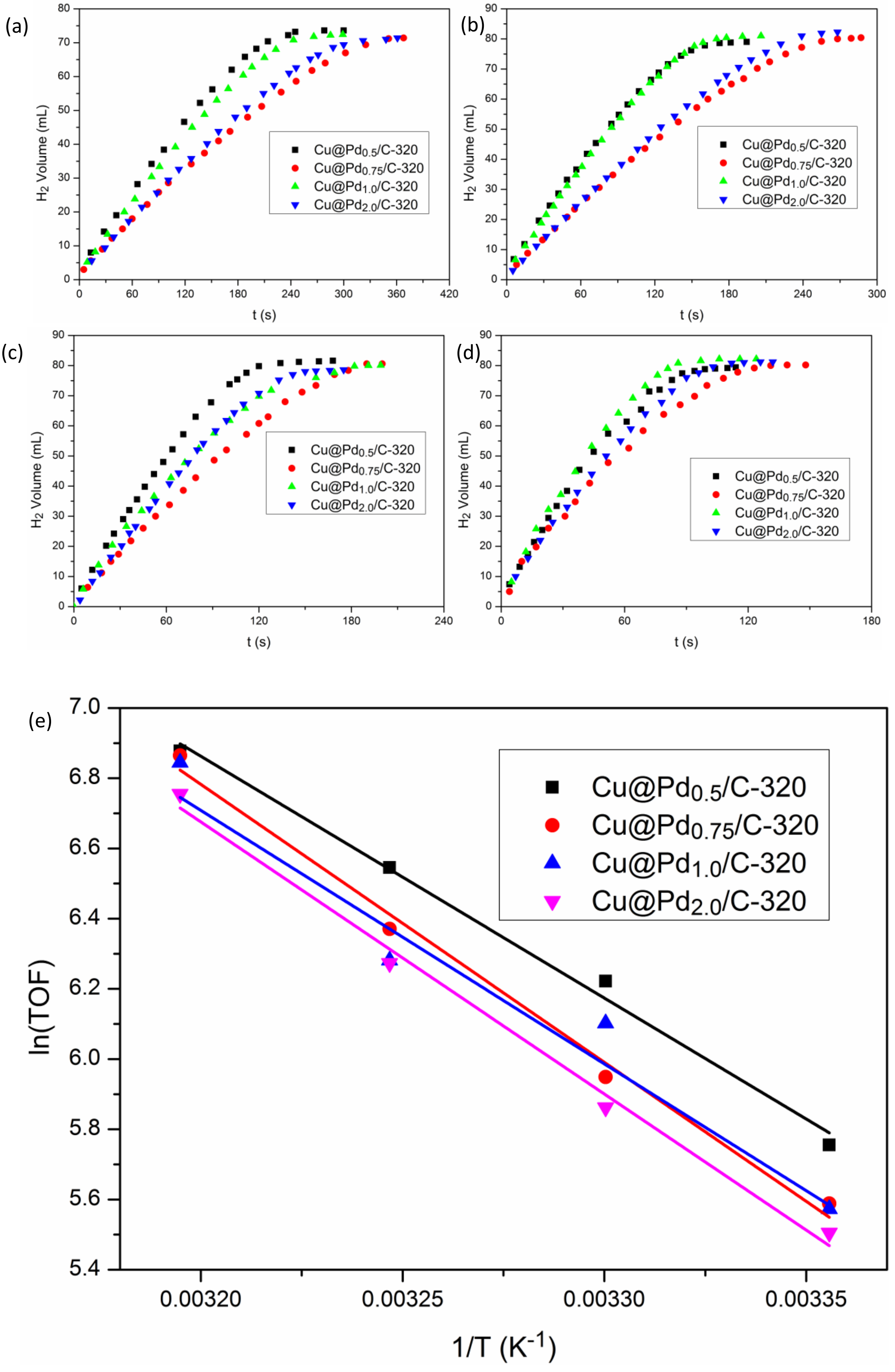


Figure S5. Plots of hydrogen evolution from ammonia borane vs time on Cu@Pd/C-320 at 298 K (a), 303 K (b), 308 K (c), 313 K (d) and the corresponding Arrhenius plot (e).

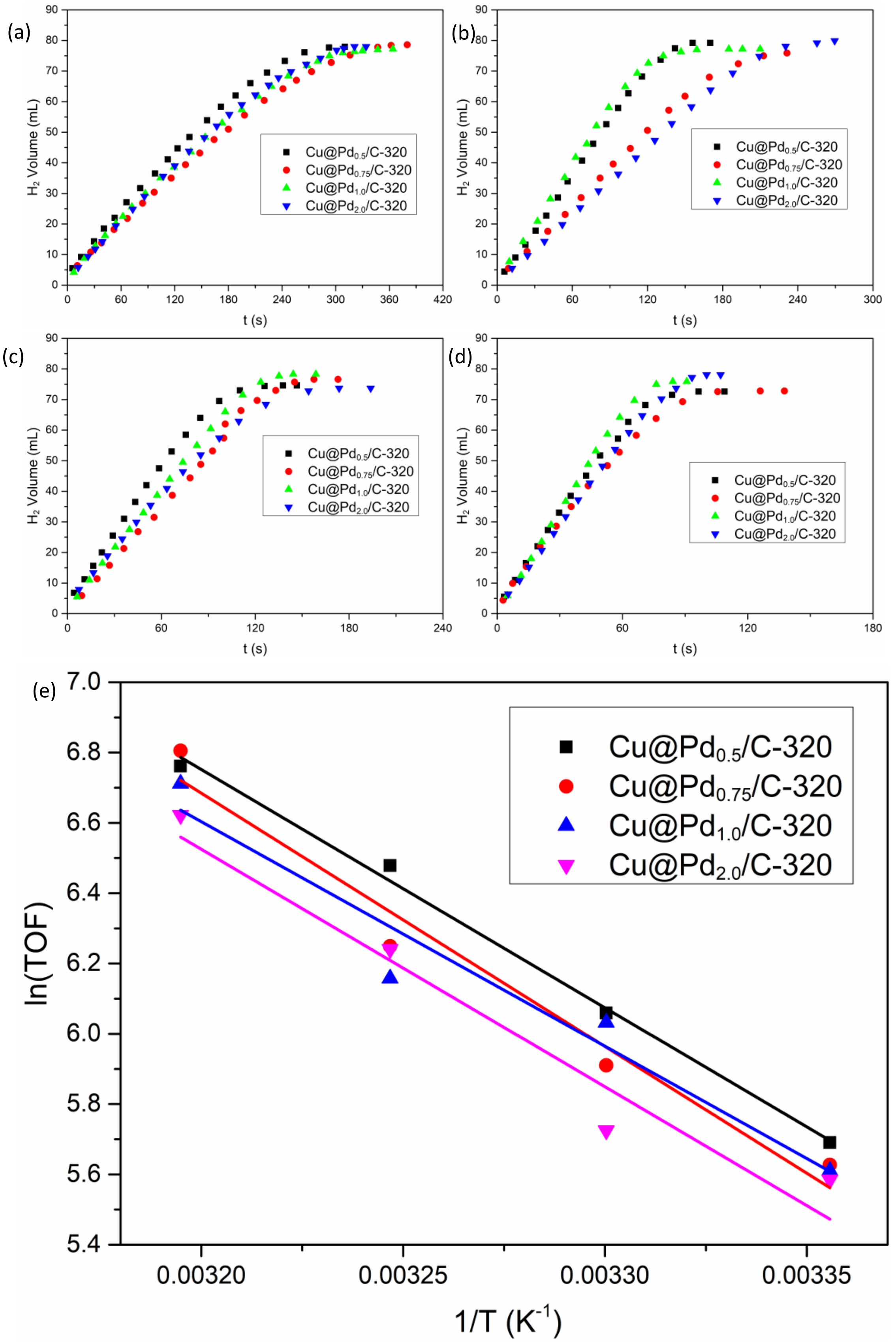


Figure S6. Plots of hydrogen evolution from ammonia borane vs time on Cu@Pd/C-320 at 298 K (a), 303 K (b), 308 K (c), 313 K (d) and the corresponding Arrhenius plot (e).

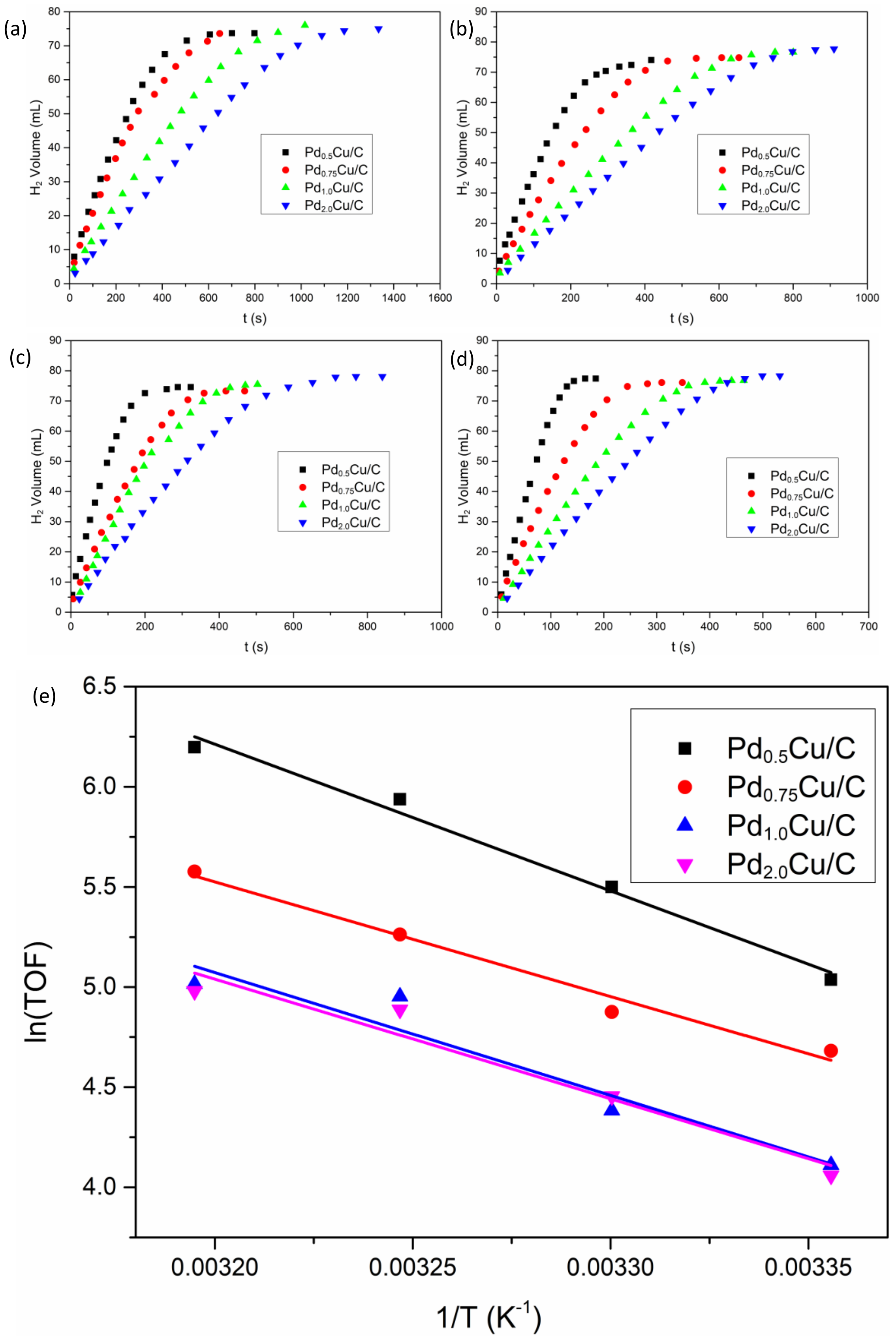


Figure S7. Plots of hydrogen evolution from ammonia borane vs time on PdCu/C at 298 K (a), 303 K (b), 308 K (c), 313 K (d) and the corresponding Arrhenius plot (e).

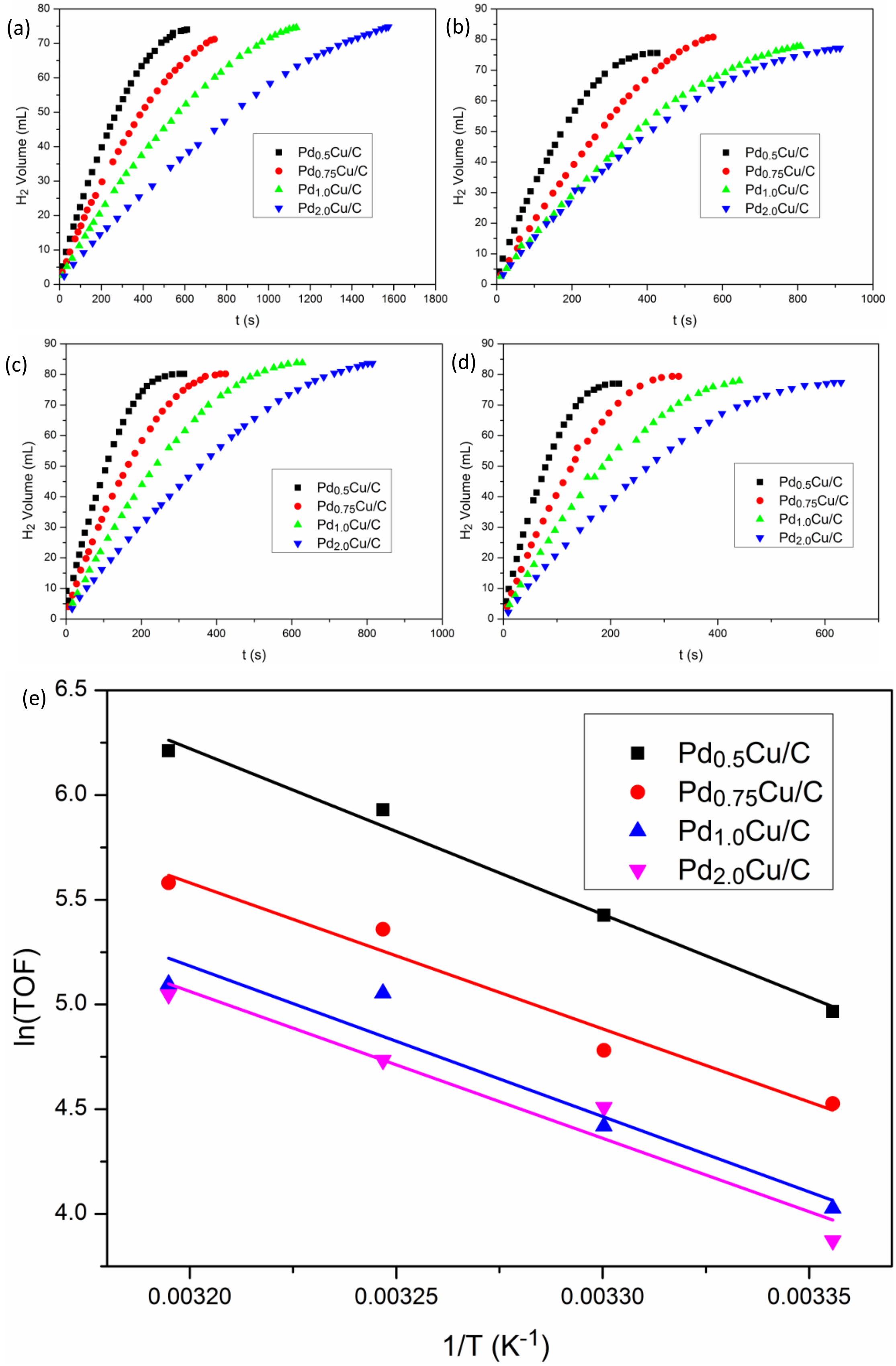


Figure S8. Plots of hydrogen evolution from ammonia borane vs time on PdCu/C at 298 K (a), 303 K (b), 308 K (c), 313 K (d) and the corresponding Arrhenius plot (e).

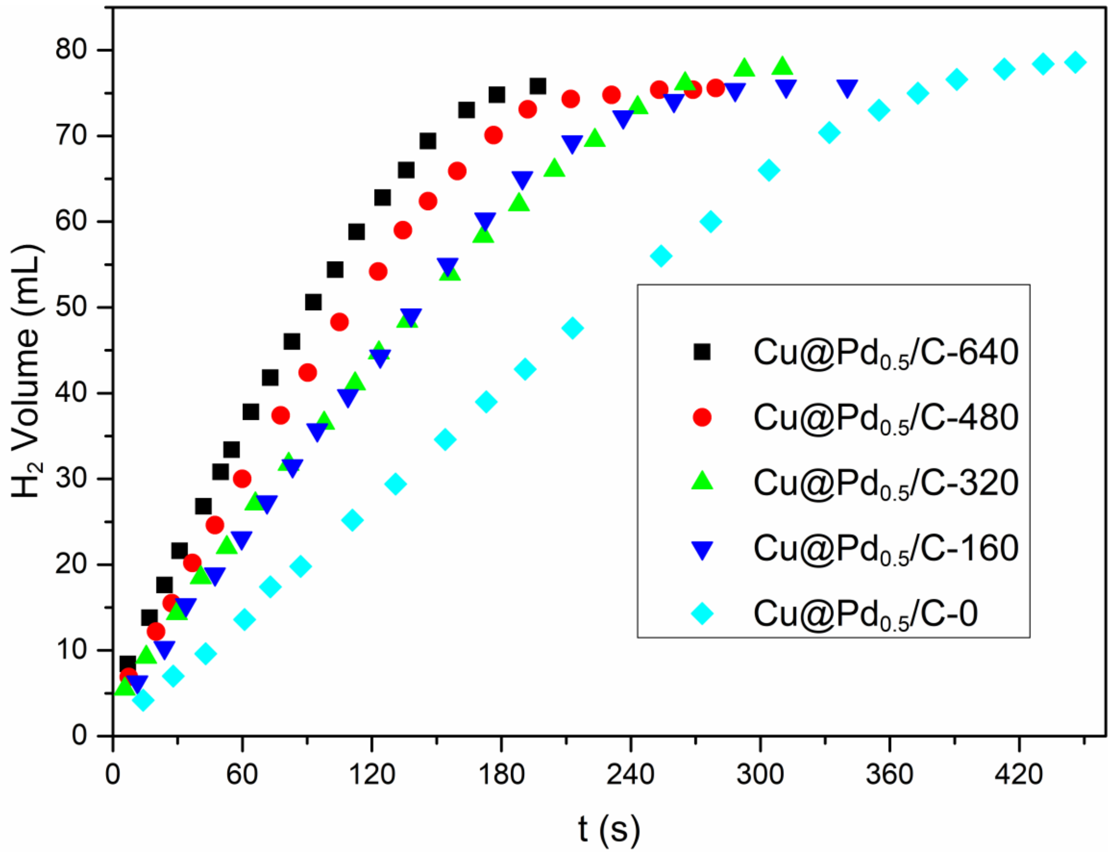


Figure S9. Plots of hydrogen evolution from ammonia borane vs time over Cu@Pd/C prepared from Cu/C with different oxidation degree.

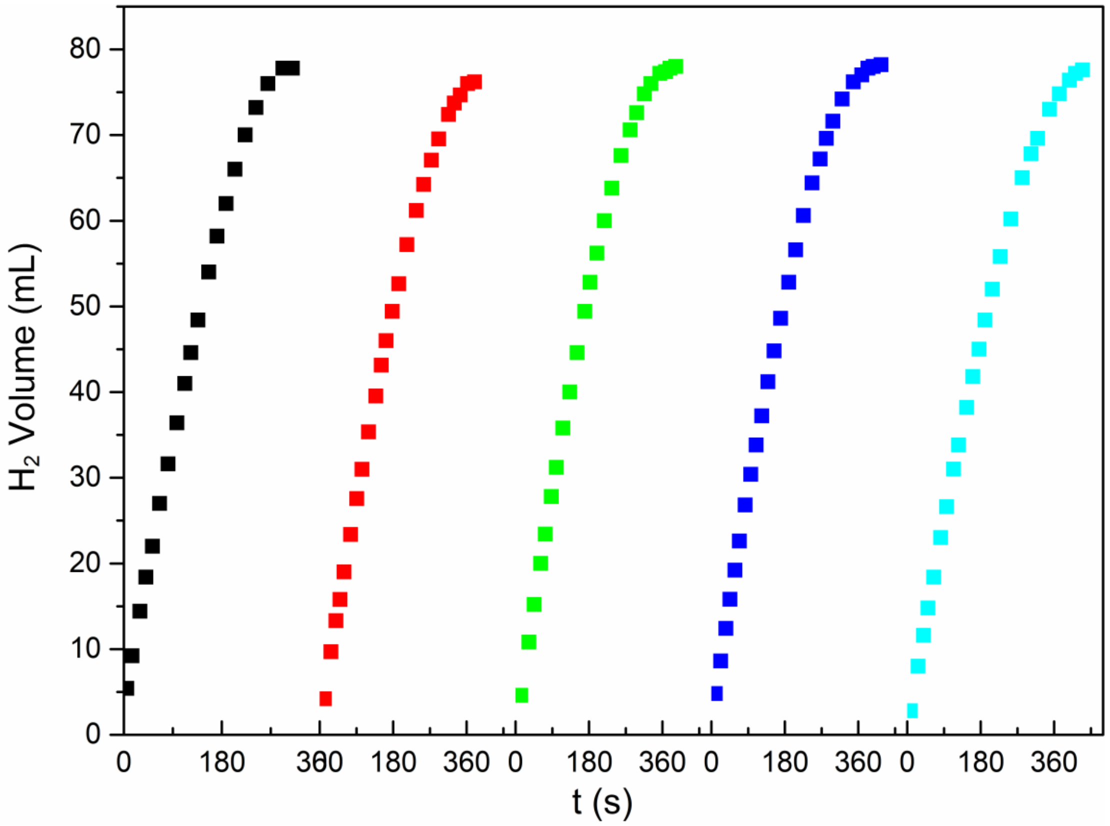


Figure S10. The stability test of Cu@Pd0.5/-320.

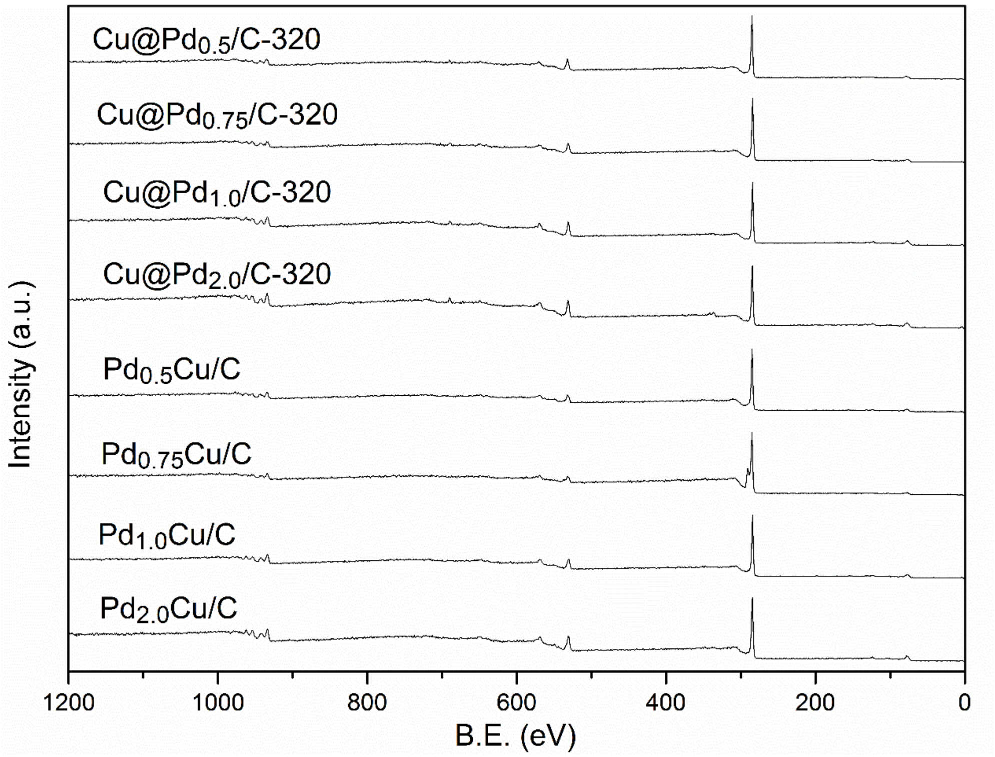


Figure S11. Survey XPS spectra of PdCu/C and Cu@Pd/C.

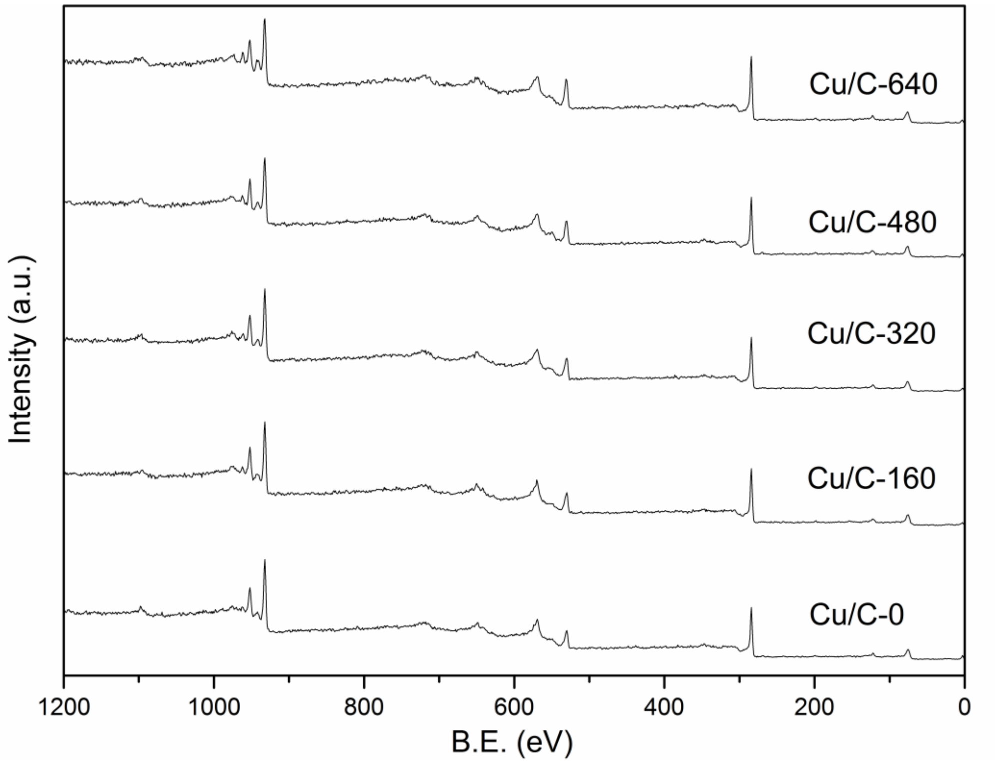


Figure S12. Survey XPS spectra of Cu/C.

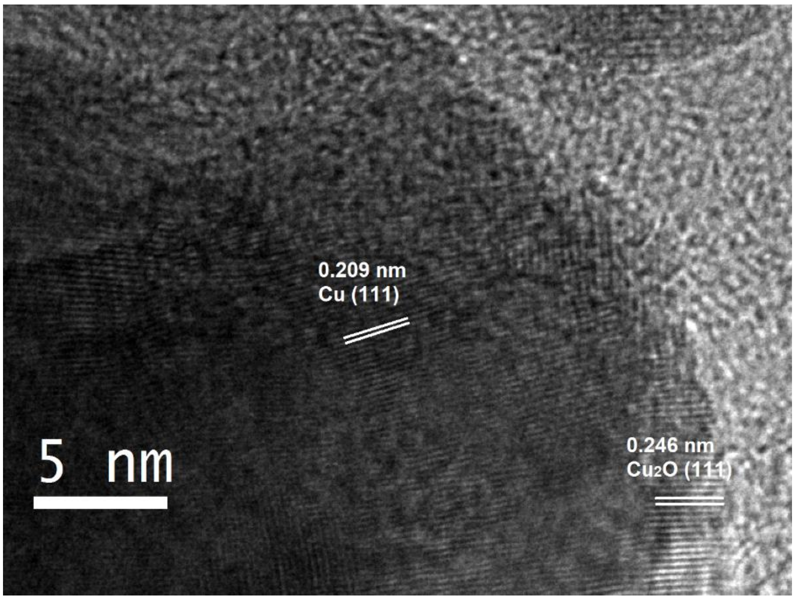


Figure S13. The HRTEM image of Cu@$Pd_{0.5}$/C-320-R.

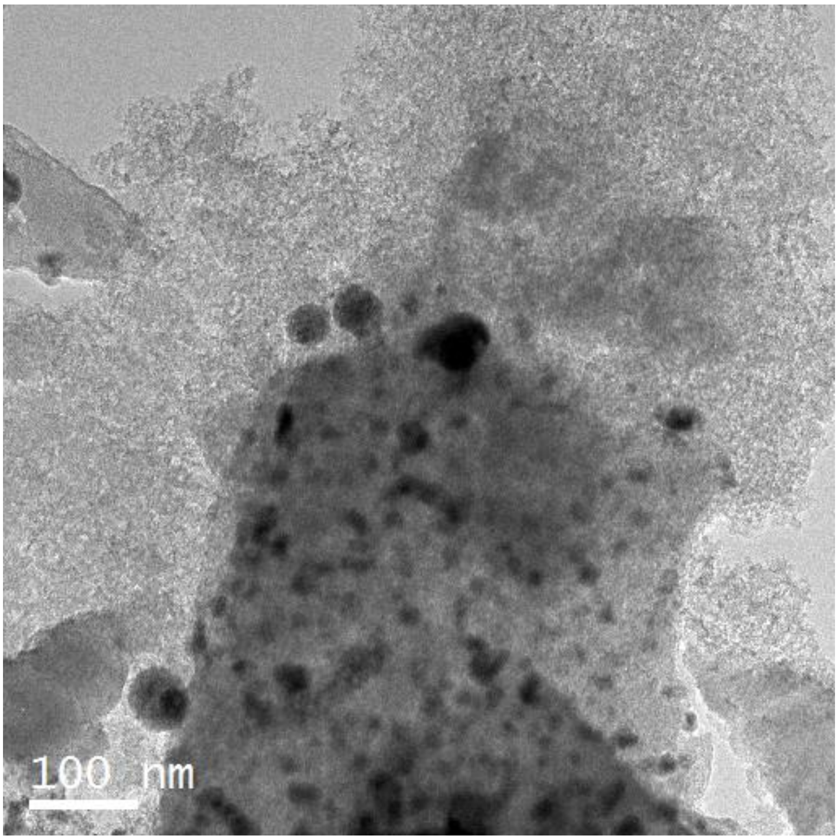


Figure S14. TEM image of Cu@$Pd_{0.5}$/C-320-R.

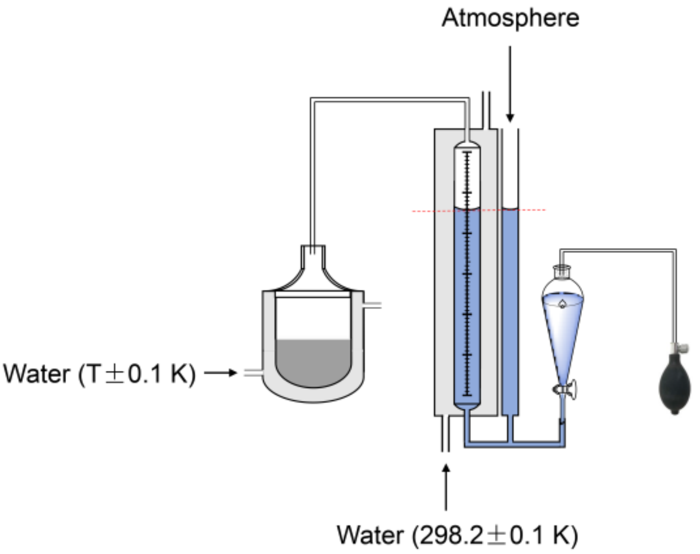


Figure S15. The schematic diagram of apparatus for catalytic hydrolysis of AB.

Table S1 The content of Pd and Cu in the sample determined by ICP-OES.

| Catalysts | Content of Pd (wt%) | Content of Cu (wt%) |
|---|---|---|
| Cu@$Pd_{0.5}$/C-0 | 0.366 | 6.852 |
| Cu@$Pd_{0.5}$/C-160 | 0.372 | 5.760 |
| Cu@$Pd_{0.5}$/C-320 | 0.376 | 6.153 |
| Cu@$Pd_{0.5}$/C-480 | 0.391 | 6.763 |
| Cu@$Pd_{0.5}$/C-640 | 0.393 | 6.404 |
| Cu@$Pd_{0.75}$/C-320 | 0.557 | 5.561 |
| Cu@$Pd_{1.0}$/C-320 | 0.905 | 5.582 |
| Cu@$Pd_{2.0}$/C-320 | 1.744 | 5.256 |
| $Pd_{0.5}$Cu/C | 0.431 | 5.217 |
| $Pd_{0.75}$Cu/C | 0.653 | 6.353 |
| $Pd_{1.0}$Cu/C | 1.062 | 5.805 |
| $Pd_{2.0}$Cu/C | 1.910 | 5.418 |
| Cu@$Pd_{0.5}$/C-320-R | 0.341 | 4.705 |
